# Supplementary figures and images for: Sleep disrupts complex spiking dynamics in the neocortex and hippocampus
Source: PLoS One. 2023 Aug 17;18(8):e0290146. doi: 10.1371/journal.pone.0290146 (PMC10434889; doi:10.1371/journal.pone.0290146)

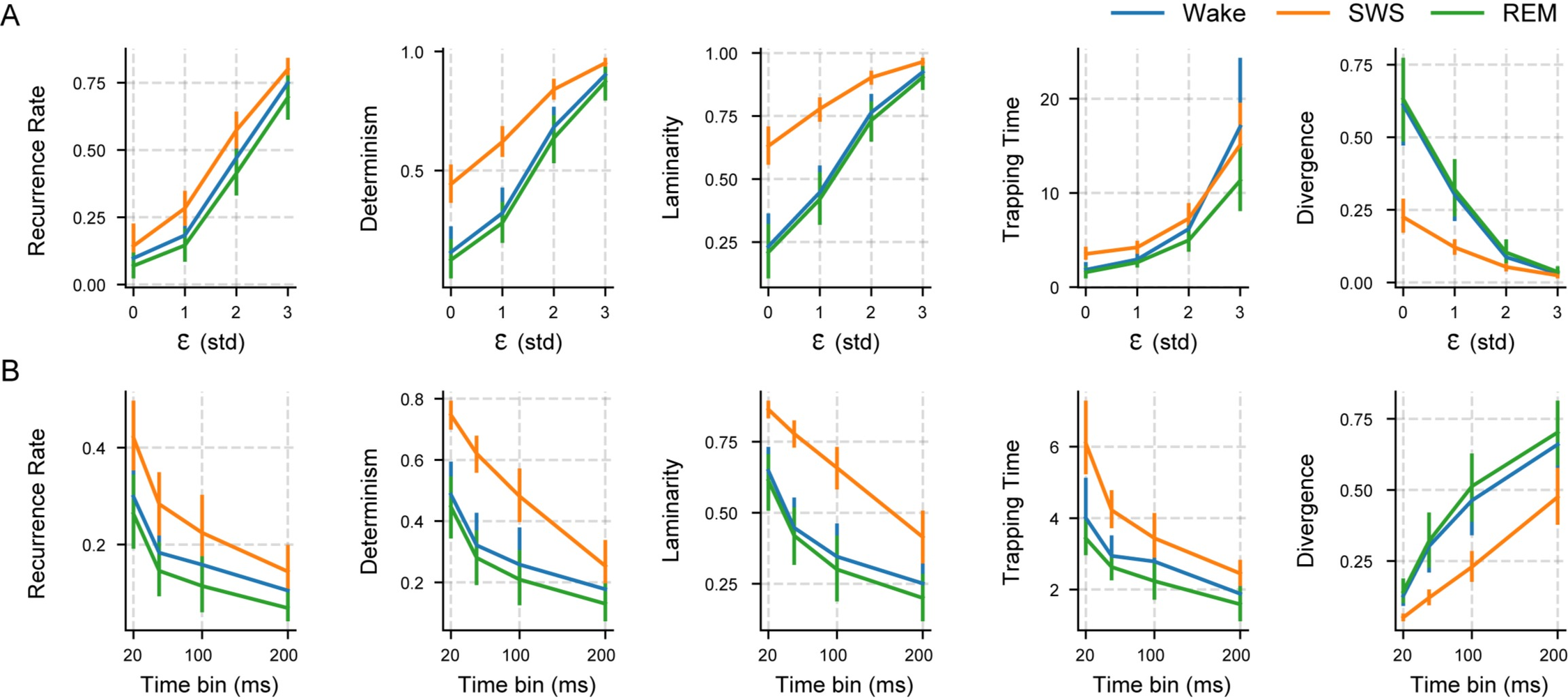

Supplement: S1 Fig — A RQA metrics for different tolerance levels ϵ defining recurrence in phase space. We vary ϵ from 0 std to 4 std of the population firing counts. Setting ϵ to 0 means that a recurrence occurs between two times for the exact same neuronal firing pattern. The time bin is kept fix at 50 ms. B RQA metrics for different time binning of the population activity. Time bins are changed from 20 ms to 200 ms in order to define the firing counts for each neuron. The ϵ is kept fix at 1 std. The mean and its corresponding 95% confidence intervals are shown for each plot. (TIF) [file pone.0290146.s001.tif]

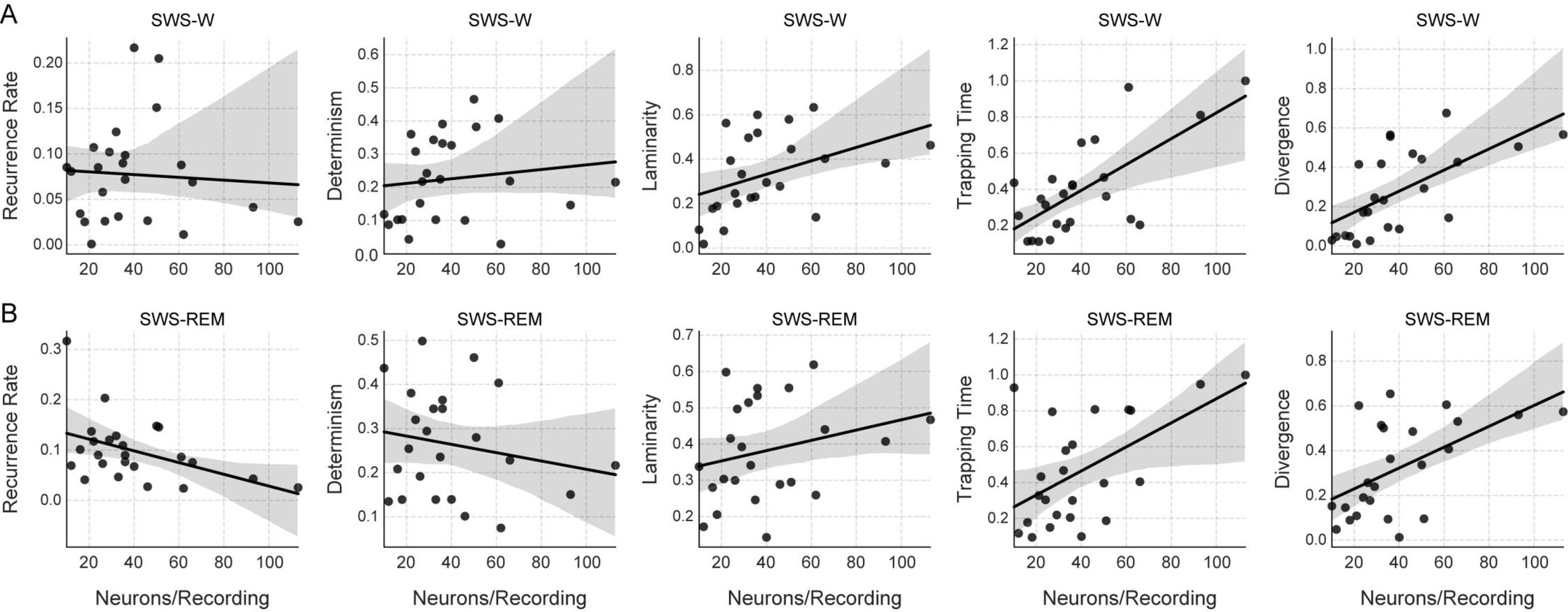

Supplement: S2 Fig — Absolute RQA differences between states as a function of the number of simultaneously recorded neurons. Each dot shows a recording session while the solid line the linear regression estimate with its 95% confidence interval. A shows the SWS-Wake difference, while B the SWS-REM difference. (TIF) [file pone.0290146.s002.tif]

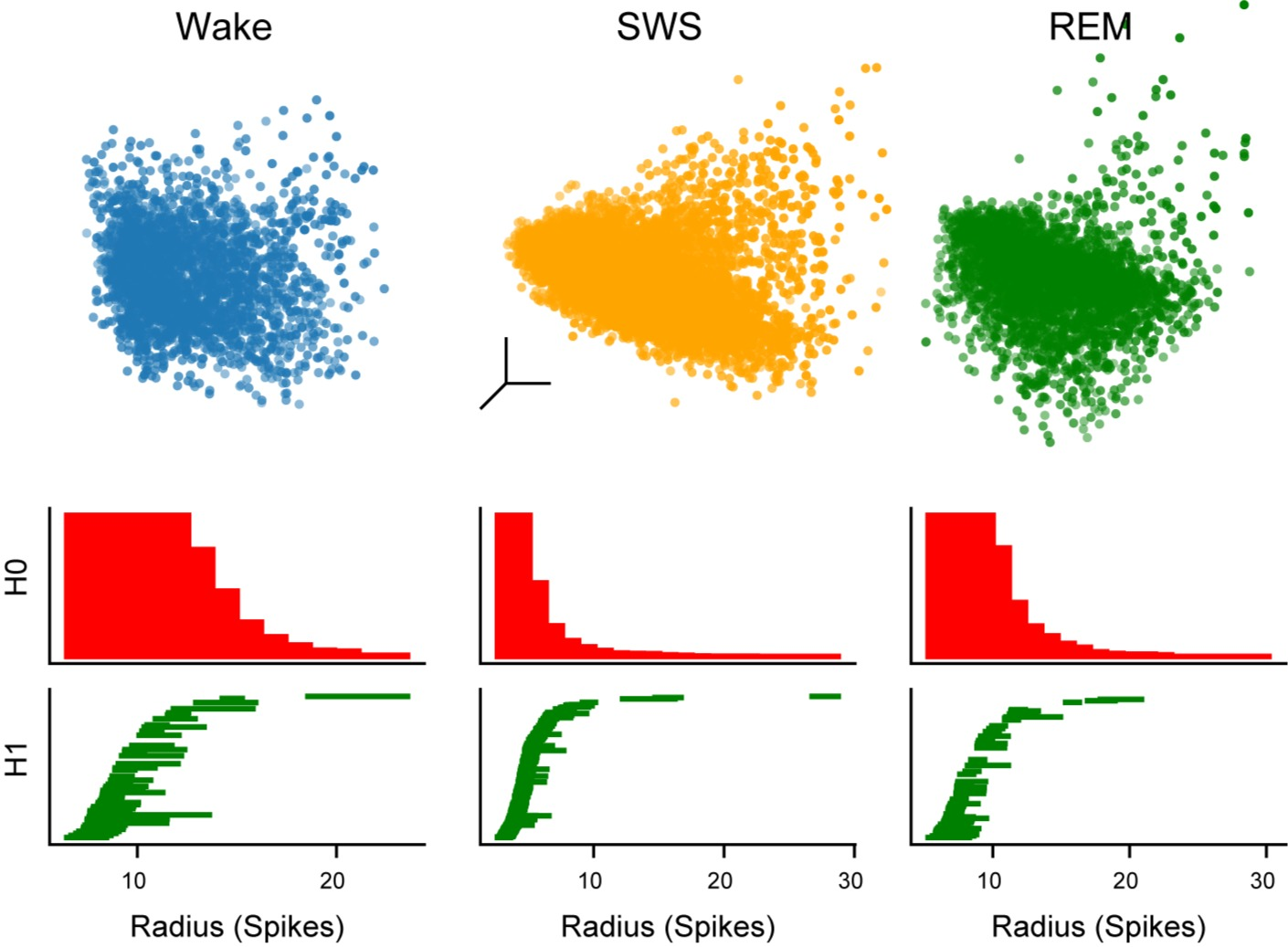

Supplement: S3 Fig — Top panels: Point clouds obtained after dimensionality reduction. A representative animal is shown during Wake, SWS and REM sleep. Bottom panels: Betti 0 (HO) and Betti 1 (H1) barcodes for the same animal shown in the top panel. The length of each bar shows the level of persistence of each Betti 0 and 1 component. (TIF) [file pone.0290146.s003.tif]

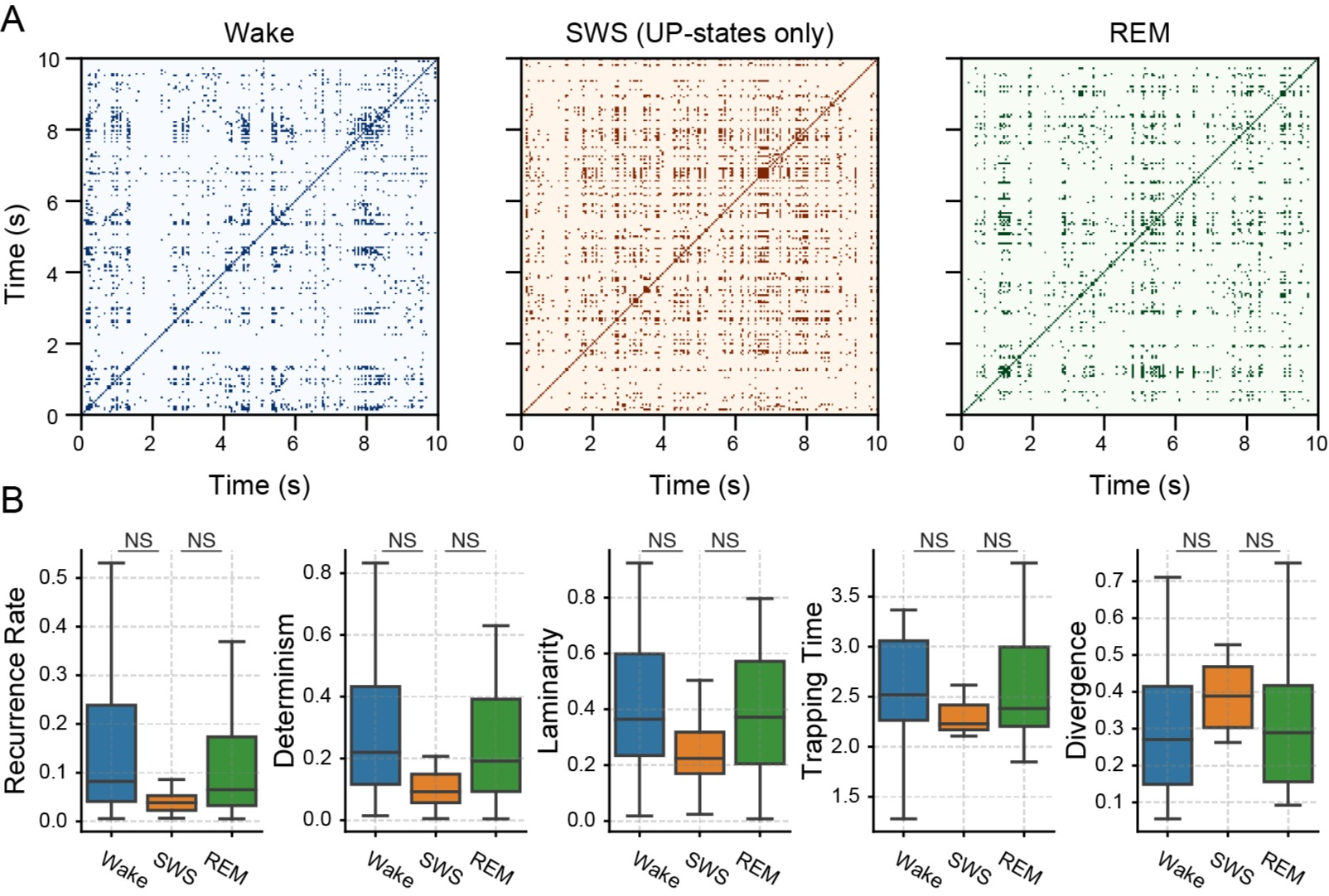

Supplement: S4 Fig — A Recurrence plots constructed from a 10s interval of the population activity using. B 5 RQA metrics for the sleep-wake states; boxplots show results from the pool of 24 sessions across 12 animals (outliers are not shown). (TIF) [file pone.0290146.s004.tif]

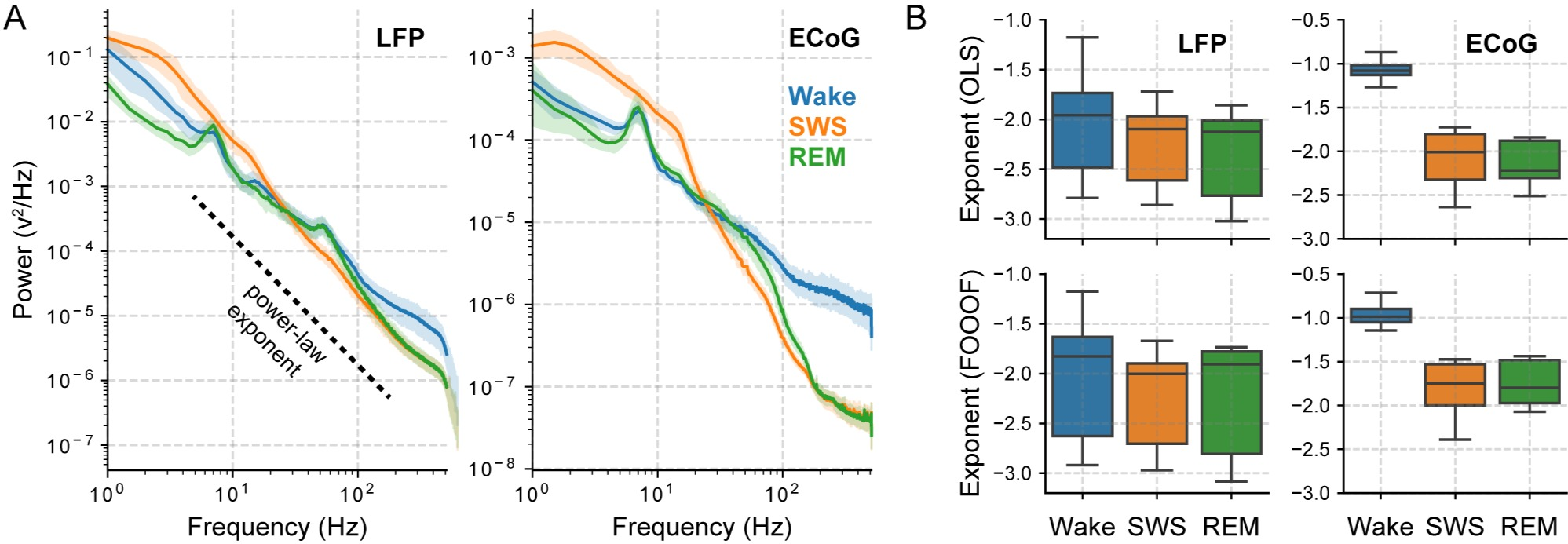

Supplement: S5 Fig — A LFP [ECoG] recordings coming from the frontal cortex [M1 cortex] during the states of Wake, SWS and REM sleep. The mean and its corresponding 95% confidence intervals are shown for each plot. B Power spectrum exponents calculated through ordinary least-squares fit on a log-log scale (OLS) or through the FOOOF parametrized spectra (FOOOF) [78] which only includes the aperiodic component. (TIF) [file pone.0290146.s005.tif]

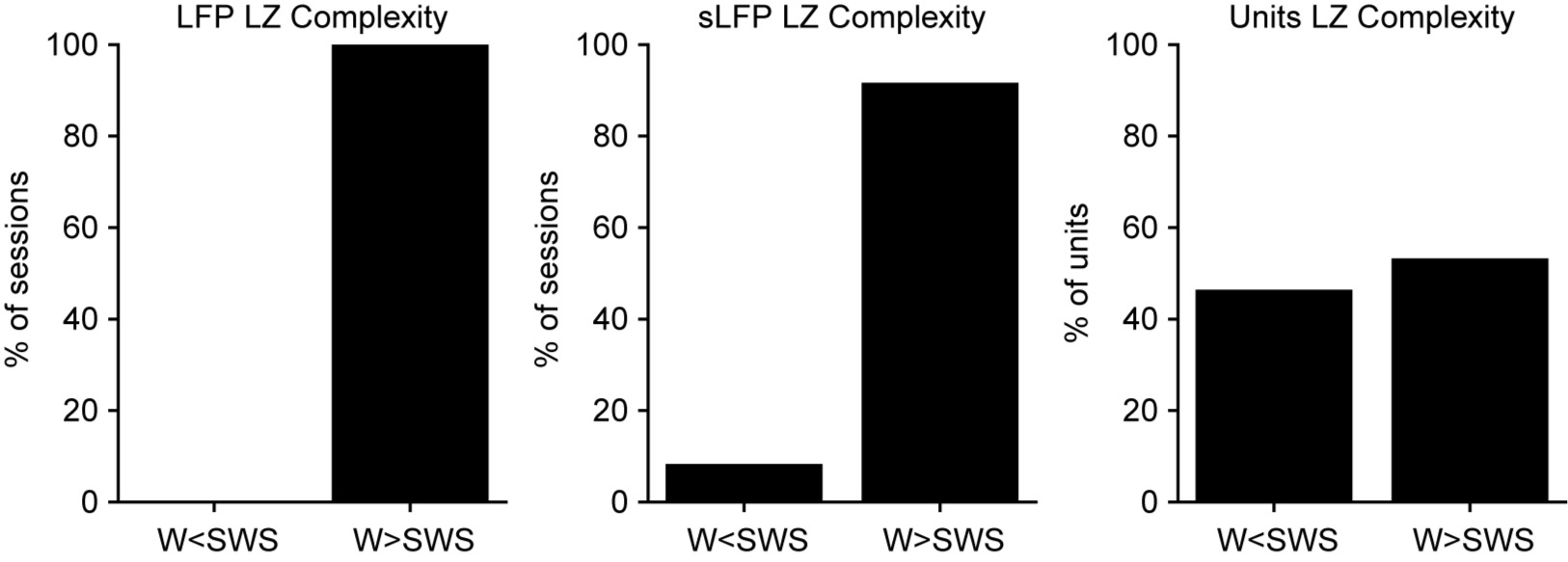

Supplement: S6 Fig — Lempel-Ziv Complexity of single neuron firing pattern between Wake and SWS. Each bar shows the total number of neurons or sessions whose temporal complexity decreased or increased during sleep. Left: LFP recordings. Middle: sLFP recordings- Right: Single unit recordings. (TIF) [file pone.0290146.s006.tif]
