# Supplementary material for: Sleep disrupts complex spiking dynamics in the neocortex and hippocampus
Source: PLoS One. 2023 Aug 17;18(8):e0290146. doi: 10.1371/journal.pone.0290146 (PMC10434889; doi:10.1371/journal.pone.0290146)
Supplement: S1 Text — (PDF) [file pone.0290146.s007.pdf]

Supplementary Methods

Persistent homology

We employ similar procedures as in [21, 53] to study the topology of the neural manifolds during the states of sleep and wakefulness. We bin the spike data in $100\,ms$ to find the firing counts and then reduce the dimensionality to a 3D representation by means of the `isomap` algorithm. We then quantify persistent homology by means of the `ripser` python3 package, limiting the analysis to Betti number 0 and 1, selecting the most persistent Betti 0 and 1 components for each session in each state to compare between conditions. Betti 0 represents the number of connected components obtained, Betti 1 shows the number of one-dimensional circular holes, and Betti 2 represents the number of two-dimensional holes or cavities.

ECoG recordings

All ECoG recordings came from our previous publication [9]. 12 Wistar adult rats were employed and recorded during their sleep-wake cycle through a stainless-steel screw electrode placed above the motor cortex (M1, reference electrode placed above the cerebellum). These recordings are available upon reasonable request to the authors.
