# Supplementary material for: Sleep disrupts complex spiking dynamics in the neocortex and hippocampus
Source: PLoS One. 2023 Aug 17;18(8):e0290146. doi: 10.1371/journal.pone.0290146 (PMC10434889; doi:10.1371/journal.pone.0290146)
Supplement: S1 File — (ZIP) [file pone.0290146.s008.zip › SupTables.pdf]

Table 1. Statistical comparisons for RQA in the neocortex

Metric	W-SWS-REM<br>p	W-SWS<br>p, Cohen's D	REM-SWS<br>p, Cohen's D
RR	1.8×10^{-8}	4.6×10^{-4}, 0.7	9.7×10^{-9}, 1.0
DET	1.1×10^{-8}	7.9×10^{-5}, 1.6	9.7×10^{-9}, 2.0
LAM	3.3×10^{-9}	2.2×10^{-5}, 1.7	4.0×10^{-9}, 1.9
TT	1.8×10^{-8}	4.2×10^{-5}, 1.2	2.3×10^{-8}, 1.5
DIV	9.5×10^{-8}	7.9×10^{-5}, 1.1	1.2×10^{-7}, 1.2

Table 2. Statistical comparisons for RQA in the hippocampus

Metric	W-SWS-REM<br>p	W-SWS<br>p, Cohen's D	REM-SWS<br>p, Cohen's D
RR	1.5×10^{-3}	1.8×10^{-2}, 2.4	1.3×10^{-3}, 1.6
DET	8.0×10^{-4}	3.6×10^{-2}, 4.4	5.3×10^{-4}, 3.2
LAM	8.0×10^{-4}	3.6×10^{-2}, 5.2	5.3×10^{-4}, 3.5
TT	8.0×10^{-4}	3.6×10^{-2}, 2.9	3.5×10^{-4}, 2.5
DIV	1.5×10^{-3}	1.8×10^{-2}, 2.9	1.3×10^{-3}, 2.4

Table 3. Statistical comparisons for the complexity metrics on real LFPs, shown in Fig 4D

Metric	W-SWS-SWS(UP)-REM<br>p	W-SWS<br>p, Cohen's D	REM-SWS<br>p, Cohen's D	SWS-SWS(UP)<br>p, Cohen's D	W-SWS(UP)	REM-SWS(UP)
Sample Entropy	3.0×10^{-9}	1.0×10^{-3}, 0.72	5.3×10^{-10}, 2.42	3.0×10^{-3}, 1.05	0.65, 0.006	1.0×10^{-3}, 1.57
Permutation Entropy	1.7×10^{-11}	1.0×10^{-4}, 1.24	2.2×10^{-12}, 1.69	2.8×10^{-4}, 1.01	0.73, 0.31	7.9×10^{-4}, 1.24
Lempel-Ziv Complexity	1.65×10^{-14}	1.4×10^{-3}, 3.31	3.0×10^{-6}, 3.65	4.9×10^{-15}, 6.47	3.0×10^{-6}, 2.80	1.4×10^{-3}, 1.80

Table 4. Statistical comparisons for the complexity metrics on synthetic LFPs, shown in Fig 4D

Metric	W-SWS-SWS(UP)-REM p	W-SWS p, Cohen's D	REM-SWS p, Cohen's D	SWS-SWS(UP) p, Cohen's D	W-SWS(UP)	REM-SWS(UP)
Sample Entropy	9.3×10^{-8}	1.0×10^{-4}, 1.35	1.1×10^{-4}, 1.52	7.1×10^{-8}, 1.61	0.14, 0.29	0.14, 0.29
Permutation Entropy	2.4×10^{-9}	1.0×10^{-6}, 0.46	1.0×10^{-4}, 0.81	4.6×10^{-9}, 1.27	0.31, 0.57	0.05, 0.57
Lempel-Ziv Complexity	1.7×10^{-9}	2.3×10^{-5}, 0.76	2.6×10^{-5}, 1.0	1.1×10^{-9}, 2.00	0.06, 0.76	0.06, 0.77
