# Supplementary material for: Sleep disrupts complex spiking dynamics in the neocortex and hippocampus
Source: PLoS One. 2023 Aug 17;18(8):e0290146. doi: 10.1371/journal.pone.0290146 (PMC10434889; doi:10.1371/journal.pone.0290146)
Supplement: Attachment — Submitted filename: PONE-D-23-12688_review.pdf [file pone.0290146.s009.pdf]

In this paper, the authors analyze in-vivo recordings from frontal cortical areas and from dorsal CA1 of rats, containing neuronal activity during wakefulness, slow-wave sleep (SWS) and rapid-eye-movement sleep (REM). The authors characterize the dynamics in the phase-space of neuron's activity by quantifying the amount of recurrence. The authors found that SWS have less complex dynamics compared to awake and REM states, and that this difference can be explained by the presence of DOWN states. Next, they complete this study with analysis on local field potentials (LFPs) and spike avalanches. Finally, they propose a critical branching model to explain their results. The results are interesting and the structure of the manuscript is overall clear. An important point of the manuscript is the link between the level of complexity observed at the macroscopic level, and the differences in the neuronal activity during each state. However, I find that some of the main findings are poorly described, and parts of the text are difficult to understand. I describe here my points of concern.

Major comments:

1. The results in Figure 1 are poorly described. The authors used several different measures of complexity, and I believe the authors should improve the description of these measures, and don't list them twice in the text. Together with this, I suggest the authors should improve the description of the possible recurrence plots in Panel C, and justify why these artificial examples are important to understand the following results.

2. In general, the text in the results section can be improved. For example, in page 5: the last two paragraph are repeated, with few words being replaced. The authors should decide which version to keep. Another example, in page 3: the sentence *"An attractor is evidenced as a manifold that attracts different trajectories of the system to the same region of the phase-space; the more convoluted (fractal) the attractor is, the higher the temporal complexity of its trajectories"* and the following sentence should be moved somewhere else. If it is there to justify the use of RQA, then I believe there is no need for it.

3. In Figure 4, I don't understand the need to use surrogate LFP instead of just using the LFP from the data. The authors first justify the use of sLFP as *"The motivation behind this method is that it allows to precisely control the sources which dictate the field potential and avoid the influence of any external variable not directly related to spiking activity*". Then, they show in panel C how the sLFP is similar to the LFP, and in panel D that the entropy methods they used lead to very similar results when applied to either LFP or sLFP. This, to me, suggest that there is no real difference between the two types of signals in terms of complexity. However, the results in panel E support the main claim of the paper, but they are shown only for the sLFP. It is important to see that these results hold true also for the LFP data. I suggest the authors to apply the analysis of panel E on the raw LFP, to move the results on the sLFP in the supplementary, and to keep the results on the raw data LFP as the main point. Otherwise, give an

explanation on why the raw LFP cannot be analysed as in panel E.

4. The authors referred to some supplementary figures (Figure S4, S5 and S6) only in the Discussion section. I believe the authors should refer to those figures in the Result section.

Minor comments:

1. I find that all the figures of the paper are too small compared to the text. One needs to pretty much zoom in to be able to see and understand the results. I am not aware if this problem can be solved during formatting. I suggest that the authors update the figures to increase their readability.

2. I believe that the average reader is not necessarily familiar with the concept of “complexity”. I suggest the author to better define the notion of the complexity of a neuronal signal in the text, especially the abstract and the introduction.

3. Page 5: *“The square-shaped recurrences appearing during SWS in Fig.2 can be generated by two possible mechanisms. Either the neurons remain constantly active for a period of time, or they remain silent (null firing counts) and give rise to a trajectory in the origin of the phase space. Next, we show that the latter is true and is mainly due to DOWN states“.* I do not understand why a squared-shaped recurrence could not be given by some neuron firing and some remaining silent, as along as they keep this activity for a period of time.

4. Figure 6: I suggest the authors should show also some metrics of variation around the mean, and consider doing a statistical analysis to quantify the significance of the difference in the models.
